# Lower Prealbumin and Higher CRP Increase the Risk of Voriconazole Overexposure and Adverse Reactions

**DOI:** 10.7759/cureus.46107

**Published:** 2023-09-28

**Authors:** Liangmo Lin, Xiangjun Fu, Mianhui Hong

**Affiliations:** 1 Pharmacy, Hainan General Hospital, Haikou, CHN; 2 Hematology, Hainan General Hospital, Haikou, CHN

**Keywords:** adverse reactions, concentration, crp, prealbumin, voriconazole

## Abstract

Background: Voriconazole (VRZ) is a commonly used antifungal drug. However, the drug has nonlinear metabolic kinetic characteristics. Many factors can affect the plasma drug concentration, thus affecting the safety and effectiveness of VRZ.

Objective: The aim of this study is to characterize the correlation between prealbumin (PA) or CRP and VRZ overexposure and adverse reactions.

Methods: Patients who received VRZ as a treatment and performed therapeutic drug monitoring (TDM) were included. Biomarkers and combined medications were analyzed to find out factors that were related to VRZ trough concentrations (C_min_) and overexposure (C_min _>5.0 mg/L). Receiver operating characteristic (ROC) curves were used to determine the cut-off levels. Patients were divided into three groups according to different PA and CRP levels. Then, the incidence rate of VRZ adverse reactions between groups was analyzed.

Results: A total of 123 patients were included in the study. PA was negatively correlated, while CRP was positively correlated with VRZ concentrations. Lower PA or higher CRP was related to VRZ overexposure with a cut-off level of 145.5 mg/L and 102.23 mg/L, respectively. Patients in Group 2 (PA <145.5 mg/L and CRP >102.23 mg/L) had an incidence rate of adverse reactions up to 70.27%, while the incidence rates in Group 1 (PA >145.5 mg/L and CRP <102.23 mg/L) and Group 3 (PA <145.5 mg/L and CRP <102.23 mg/L or PA >145.5 mg/L and CRP >102.23 mg/L) were 15.38% and 32.43%, respectively.

Conclusions: PA and CRP were both related to VRZ concentrations and overexposure. The risk of VRZ overexposure and adverse reactions significantly increased in patients with PA <145.5 mg/L and CRP >102.23 mg/L at the same time.

## Introduction

Voriconazole (VRZ) belongs to the triazole antifungal agents used as the first-line treatment for invasive aspergillosis [1]. Additionally, VRZ is widely employed for the treatment and prophylaxis of invasive fungal diseases (IFDs). Due to its nonlinear pharmacokinetics, VRZ exhibits highly variable metabolism and a narrow therapeutic range among patients [2]. Consequently, therapeutic drug monitoring (TDM) is recommended to optimize individualized VRZ medication. The Chinese Pharmacological Society suggests a target range for VRZ trough concentrations (C_min_) between 0.5 mg/L and 5.0 mg/L [3].

Various factors have been reported to influence the pharmacokinetics of VRZ. The drug is extensively metabolized through cytochrome P450 (CYP450) enzymes, particularly CYP2C19. Patients classified as CYP2C19 poor metabolizers (PM) may face a higher risk of VRZ overexposure or toxicity [4-5]. Furthermore, hepatic dysfunction and certain co-administered medications also affect VRZ metabolism [6-7]. Recent studies have revealed that inflammatory status, assessed by CRP, serves as a significant risk factor contributing to elevated VRZ concentrations and subsequent adverse reactions [8-10]. Inflammation appears to weaken the activity of CYP450 enzymes, thus reducing VRZ metabolism [8]. However, the precise CRP level that impacts VRZ pharmacokinetics remains uncertain. Additionally, the potential correlation between other biomarkers reflecting nutritional status and liver function, such as prealbumin (PA), and further research was needed on the relationship between PA and VRZ. The primary objective of this study is to identify factors associated with VRZ overexposure and adverse reactions within a cohort of Chinese patients undergoing TDM during VRZ treatment.

This article was previously published on Research Square on October 4, 2022.

## Materials and methods

Patients

Patients who received VRZ for the prevention or treatment of IFDs between January 2020 and August 2022 were enrolled in this retrospective clinical study. The inclusion criteria were as follows: (1) patients who received a maintenance dose of VRZ at 200 mg every 12 hours (q12h) with or without a loading dose of 400 mg q12h, (2) age >18 years, and (3) received TDM of VRZ during treatment, with only the first TDM result considered for the study. Exclusion criteria were as follows: (1) use of other dosing regimens, (2) pregnancy, (3) severe impairment of liver function (Child-Pugh class B and C), and (4) incomplete basic information. This study adhered to the ethical principles of the Declaration of Helsinki and received approval from the ethics committee of Hainan General Hospital in China with approval number 2021232. All methods were conducted in accordance with relevant guidelines and regulations as required by the hospital. Informed consent was obtained from all patients participating in the study.

Blood sampling and analytical assays

For patients receiving the loading dose of VRZ, the initial plasma sample was collected on the third day of treatment. In cases where no loading dose was administered, the sample was obtained on the sixth day. All samples were drawn within 30 minutes before drug administration. The plasma concentrations of VRZ were measured using the enzyme amplification immunoassay with Siemens equipment. Due to the limited availability of genetic testing in our institution, data on the CYP2C19 polymorphism were not included in this study.

Study design

The therapeutic target for VRZ trough concentrations (C_min_) was identified within the range of 0.5 to 5.0 mg/L. The medical records of each patient were thoroughly reviewed, and the following data were collected: demographic characteristics such as age, sex, body weight, and inpatient department; laboratory data including total protein (TPPOT), albumin (ALB), prealbumin (PA), alanine aminotransferase (ALT), aspartate aminotransferase (AST), total bilirubin (TBIL), direct bilirubin (DBIL), indirect bilirubin (IBIL), and CRP. Prescription information, such as delivery route, medication course, combined medication, and TDM results, was also documented. Adverse reactions caused by VRZ, including hepatotoxicity, visual abnormalities, and nervous system abnormalities, were collected for analysis. Initially, we aimed to identify risk factors, including biomarkers and combined medication, associated with VRZ C_min_ levels. Receiver operating characteristic (ROC) curves were utilized to determine the specific cut-off values of biomarkers that could lead to VRZ overexposure (C_min_ >5.0 mg/L). Subsequently, patients were categorized into different groups based on the ROC results, and the relationship between these risk factors and VRZ adverse reactions was analyzed.

Statistical analysis

Baseline data of patients' demographic characteristics were presented as mean ± standard deviation (SD), along with the maximum and minimum values. Pearson correlation analysis was employed to assess the presence of a linear relationship between continuous variables and VRZ C_min_. Binary logistic regression analysis was used to identify risk factors associated with VRZ overexposure. The ROC curve was employed to determine the optimal cut-off level for continuous variables. ANOVA was used to compare differences between patient groups. Chi-square analysis was utilized to examine the relationship between risk factors and VRZ adverse reactions. All statistical analyses were performed using SPSS Statistics version 20.0 (IBM Corp. Released 2011. IBM SPSS Statistics for Windows, Version 20.0. Armonk, NY: IBM Corp.), and a significance level of p<0.05 was considered statistically significant.

## Results

Patient clinical characteristics and VRZ regimen

A total of 123 patients undergoing VRZ treatment during the study period were included, comprising 74 males and 49 females. Among them, 64 patients were from the hematological department, 29 from the respiratory department, 9 from the intensive care unit, 9 from the rheumatology and immunization department, 8 from the emergency room, and 4 from other departments. The initial dosing regimen of VRZ was 200mg q12h, with or without a loading dose of 400mg q12h. Only the first TDM result of VRZ C_min_ was considered to minimize the impact of dose adjustments. Tables 1-2 display the patients' clinical characteristics and VRZ regimen. Notably, there was significant inter-individual variability observed in VRZ C_min_, with an average value of 4.96±2.69 mg/L, ranging from 0.19 to 12.97 mg/L. The average duration of VRZ administration was 25.46±23.34 days, ranging from 3 to 180 days.

**Table 1:** Clinical characteristics of the patients

Patient clinical characteristics	Mean±SD	Min	Max
Age (years)	54.14±17.86	18.00	90.00
Weight (kg)	57.12±10.13	40.00	80.00
TPPOT (g/L)	59.61±10.86	29.70	93.20
ALB (g/L)	29.58±6.09	16.20	62.50
PA (mg/L)	138.60±77.14	21.00	392.00
ALT (U/L)	31.21±30.25	2.00	138.7
AST (U/L)	30.70±33.15	5.10	163.80
TBIL (μmol/L)	10.51±8.07	1.97	40.10
DBIL (μmol/L)	5.28±6.59	0.25	32.10
IBIL (μmol/L)	5.87±4.01	1.15	26.82
CRP (mg/L)	103.79±83.72	2.92	327.76
VRZ C_min _(mg/L)	4.96±2.69	0.19	12.97

**Table 2:** The VRZ regimen

VRZ regimen	
Days of administration (Mean±SD.days)	25.46±23.34
With a loading dose (n, %)	79, 64.23%
Without a loading dose (n, %)	44, 35.77%
Intravenous administration (n, %)	85, 69.11%
Oral administration (n, %)	14, 11.38%
Intravenous to oral (n, %)	24, 19.51%
Combined with omeprazole (n, %)	13, 10.57%
Combined with rabeprazole (n, %)	8, 6.50%
Combined with pantoprazole (n, %)	42, 34.15%
Combined with tigecycline (n, %)	10, 8.13%

Relationship between a continuous variable and VRZ C_min_

**Figure 1:**
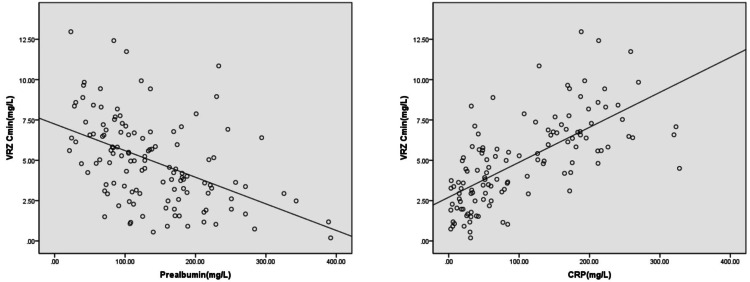
Scatter plots of PA CRP and VRZ Cmin VRZ: voriconazole, CRP: C-reactive protein

Pearson correlation analysis was employed to examine the relationship between continuous variables of patient clinical characteristics, such as weight, TPPOT, ALB, PA, ALT, AST, TBIL, DBIL, IBIL, CRP, and VRZ C_min_. The results revealed that only PA and CRP exhibited a moderate linear correlation with C_min_. No significant relationship was observed for other characteristics. The Pearson correlation coefficients (r) for PA and CRP were -0.512 and 0.549, respectively (both p=0.000). Scatter plots depicting the relationships between PA or CRP and C_min_ are presented in Figure 1.

Risk factors that lead to VRZ overexposure

**Figure 2:**
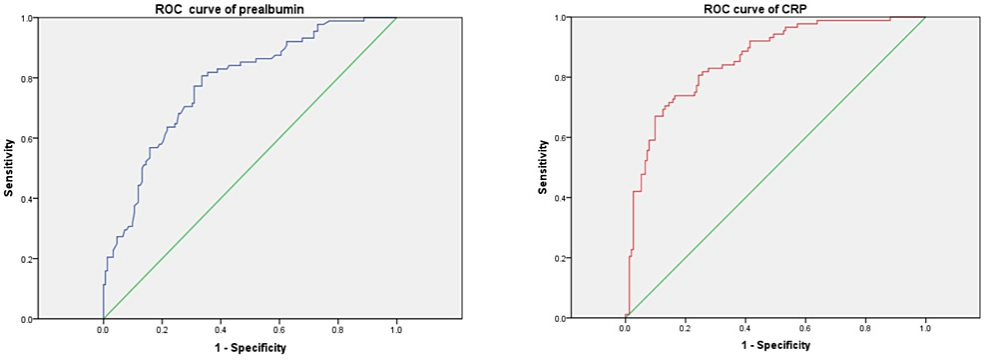
ROC curves of PA CRP and VRZ high exposure ROC: receiver operating characteristic, CRP: C-reactive protein

As per the guidelines of the Chinese Pharmacological Society, VRZ overexposure was defined as C_min_ >5.0 mg/L, and the proportion of patients with C_min_ >5.0 mg/L in this study was 47.15%. We examined the influence of PA, CRP, and combined medications such as proton pump inhibitors (PPI) and tigecycline on VRZ overexposure. The results from binary logistic regression analysis indicated that lower PA levels (OR = 0.99; 95% CI: 0.985-0.996; p = 0.001) and higher CRP levels (OR = 1.015; 95% CI: 1.010-1.020; p = 0.000) were associated with an increased risk of VRZ overexposure, while none of the combined medications demonstrated a promoting effect. Through the use of ROC curve analysis, we identified the optimal cut-off levels of 145.5 mg/L for PA and 102.23 mg/L for CRP. The area under the curve for PA was 0.779, and for CRP, it was 0.859, indicating a clear correlation with C_min_ >5.0 mg/L. The ROC curves are depicted in Figure 2.

Lower PA and higher CRP increase the risk of adverse reactions

Ten patients who received tigecycline during VRZ treatment were excluded from the next study due to tigecycline's known hepatotoxicity in clinical application. The remaining 113 patients were then divided into three groups based on their PA and CRP levels: Group 1 with PA >145.5 mg/L and CRP <102.23 mg/L, Group 2 with PA <145.5 mg/L and CRP >102.23 mg/L, and Group 3 with PA >145.5 mg/L and CRP >102.23 mg/L or PA <145.5 mg/L and CRP <102.23 mg/L. Analysis of variance revealed no significant differences in other patient clinical characteristics between the three groups, except for PA, TBIL, CRP, and C_min_. The average VRZ C_min_ in Group 2 was 6.88±2.30 mg/L, which was notably higher than that in Group 1 (2.99±1.72 mg/L) and Group 3 (4.80±2.34 mg/L). The clinical characteristics of the three groups are presented in Table 3.

**Table 3:** Patient clinical characteristics of Groups 1 to 3 TPPOT: total protein, ALB: albumin, PA: prealbumin, ALT: alanine aminotransferase, AST: aspartate aminotransferase, TBIL: total bilirubin, DBIL: direct bilirubin, IBIL: indirect bilirubin, CRP: C-reactive protein, VRZ: voriconazole

Parameter	Group 1 (n=39)	Group 2 (n=37)	Group 3 (n=37)	p-value
Age (years)	51.41±18.78	54.03±19.83	54.16±15.00	0.753
Weight (kg)	58.34±11.36	54.12±11.15	58.01±8.23	0.152
TPPOT (g/L)	62.89±11.21	55.29±11.13	59.85±8.32	0.070
ALB (g/L)	31.06±5.26	27.91±7.36	30.31±4.41	0.053
PA (mg/L)	201.48±56.52	80.38±32.87	140.46±74.11	0.000
ALT (U/L)	33.97±34.25	34.38±33.96	26.21±24.59	0.450
AST (U/L)	36.20±48.46	29.51±24.44	25.77±22.03	0.404
TBIL (μmol/L)	8.32±7.44	14.55±16.23	9.85±7.50	0.044
DBIL (μmol/L)	3.59±4.79	7.99±11.70	5.05±6.65	0.063
IBIL (μmol/L)	4.71±3.00	6.55±4.95	6.13±4.64	0.146
CRP (mg/L)	36.84±34.72	192.71±51.09	80.54±63.85	0.000
VRZ C_min _(mg/L)	2.99±1.72	6.88±2.30	4.80±2.34	0.000
Days of administration (days)	31.21±23.31	22.54±29.97	23.03±14.91	0.199

**Figure 3:**
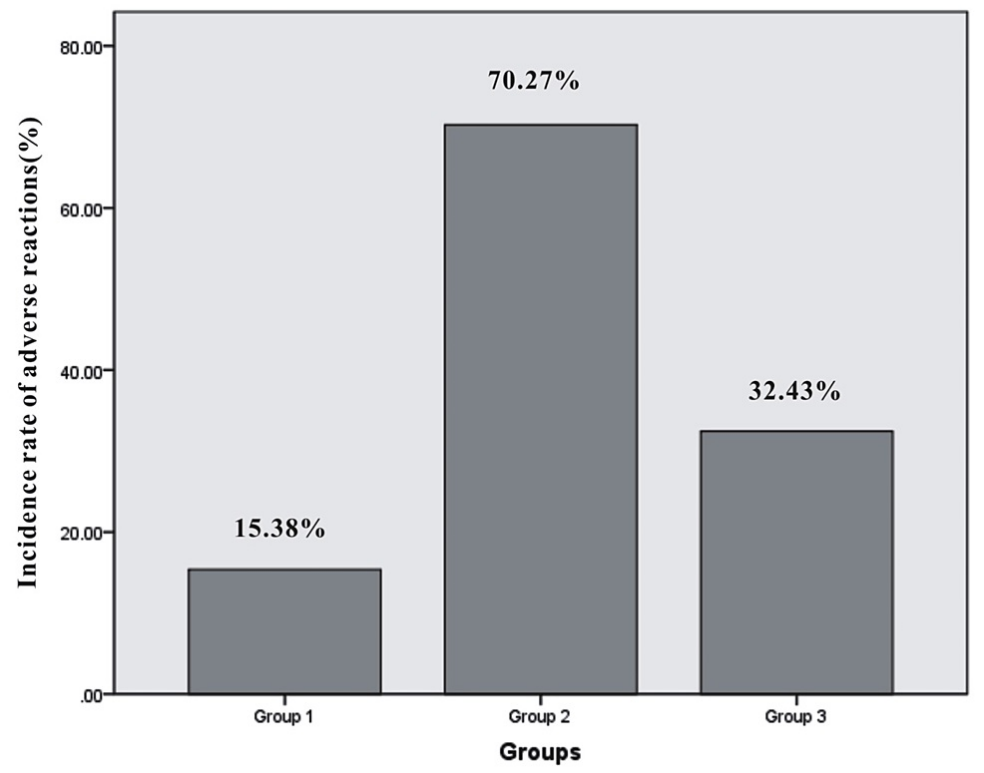
Incidence rate of adverse reactions

A total of 44 patients experienced adverse reactions to VRZ. The incidence rate of adverse reactions in Group 1 was 15.38%, all of which were related to hepatotoxicity. In Group 2, the incidence rate was remarkably higher, reaching 70.27%, with 16 cases of hepatotoxicity, 8 cases of visual abnormalities, and 2 cases of nervous system abnormalities (delirium and excitement). For Group 3, the incidence rate was 32.43%, with 12 cases of hepatotoxicity and 2 cases of visual abnormalities occurring simultaneously. Chi-square analysis revealed significant differences in the incidence rates of adverse reactions between Group 1 and Group 2 (p=0.000), as well as between Group 2 and Group 3 (p=0.001), while no significant difference was observed between Group 1 and Group 3 (p=0.081). The incidence rates and analytical results are presented in Table 4. It appears that patients in Group 2, who exhibited both lower PA and higher CRP, had a much higher C_min_ and incidence rate of adverse reactions. The incidence rates of the three groups are visually displayed in Figure 3.

**Table 4:** Incidence rate of adverse reactions of VRZ in different groups

	Incidence rate	X^2^	p-value
Group 1 vs Group 2	15.38% vs 70.27%	23.464	0.000
Group 1 vs Group 3	15.38% vs 32.43%	3.053	0.081
Group 2 vs Group 3	70.27% vs 32.43%	10.602	0.001

## Discussion

PA is commonly used to assess liver function and nutritional status, and its levels can be influenced by inflammation, aging, and malnutrition [11-12]. Due to its rapid hepatic anabolism and metabolism, PA has a shorter half-life of approximately two days, making it a more sensitive indicator for measuring liver function and malnutrition compared to ALB, which may be affected by ALB infusions or blood transfusions [13]. Low PA levels have been associated with severe infections and higher mortality rates. For instance, in a study of elderly hospitalized patients with COVID-19, it was found that older patients with lower PA levels exhibited higher neutrophil counts, lower lymphocyte counts, and impaired coagulation and liver function, leading to worse outcomes and increased all-cause mortality [14]. Another study by Ju Dong Li reported that low PA levels increased postoperative mortality and morbidity in patients undergoing hepatic resection for hepatocellular carcinoma. However, the specific relationship between PA levels and VRZ C_min_ and adverse reactions has not been extensively studied and reported.

CRP is a widely used and sensitive biomarker for inflammation. Some studies have demonstrated that CRP significantly affects PA levels. For example, Scanlan et al.'s study in stroke patients admitted to an acute rehabilitation setting revealed a significant negative association between PA and CRP (p=0.02), indicating that for every unit increase in CRP, PA decreased by 0.14 units [15]. Previous research has also explored the association between CRP and VRZ concentrations. In a case-control study involving hematological patients who underwent VRZ TDM, it was found that patients with CRP levels >96 mg/L had a 27-fold higher risk of VRZ overdose (defined as C_min_ ≥4 mg/L) compared to those with CRP levels ≤96 mg/L [8]. Another study by Veyret confirmed a positive association between CRP levels and VRZ C_min_ after adjusting for various factors, including genetics. An increase in CRP levels of 10 or 50 mg/L resulted in a 24% or 36% increase in VRZ C_min_, respectively [16]. Additionally, Veringa et al. demonstrated that VRZ metabolism was reduced during inflammation, as reflected by a decrease in the formation of VRZ-N-oxide, a metabolite of VRZ [9]. Overall, numerous studies have confirmed that elevated CRP levels are significantly associated with VRZ overexposure. Our study revealed that simultaneous lower PA and higher CRP levels could lead to excessively high VRZ C_min_ and significantly increase the risk of adverse reactions. However, our identified best cut-off level for CRP (102.23 mg/L) differed from previous studies (96 mg/L) [8], and we also highlighted the influence and best cut-off level of PA (145.5 mg/L) in VRZ overexposure. It should be noted that the definition of VRZ overexposure and concentration detection methods varied among these studies, indicating the need for future research involving multi-center studies and standardized evaluation criteria.

The role of pharmacogenetics of VRZ during inflammation remains uncertain. Gautier-Veyret et al.'s study found no significant difference in the proportion of CYP genotypes (CYP2C19, CYP3A4, or CYP3A5, considered alone or combined in a combined genetic score) between patients who experienced VRZ overdose and those who did not [8]. However, CRP levels appeared to positively influence VRZ C_min_ in patients with increased metabolic activity compared to those with normal or diminished metabolic activity [10]. Despite the higher prevalence of CYP2C19 PM in China (14.7%) compared to Europe Caucasians (2.1%) and Africa (3.7%) [16], there are currently no recommendations regarding VRZ pharmacogenetics from the Chinese Pharmacological Society due to conflicting evidence from systematic reviews and patients' preferences. The limited acceptance of VRZ pharmacogenetic testing by patients resulted in a lack of research in this area, leading to certain limitations in our study. Additionally, the analysis of variance showed differences in TBIL among the three groups, which may indicate hepatotoxicity. Further study is needed to determine the exact influence of TBIL.

In our study, we only considered the initial TDM results to minimize the impact of dose adjustments. We found that both PA and CRP were related to VRZ C_min_, with PA negatively correlated and CRP positively correlated. Other biomarkers such as weight, ALB, ALT, AST, and TBIL showed no correlation with VRZ C_min_. Logistic regression analysis indicated that lower PA and higher CRP could lead to VRZ overexposure (C_min_ >5.0 mg/L), regardless of the concurrent use of PPI or tigecycline. Due to the hepatotoxicity of tigecycline, patients receiving this drug in combination were excluded from the subsequent adverse reactions study. Patients in Group 2 (with PA <145.5 mg/L and CRP >102.23 mg/L) were more likely to experience VRZ overexposure and adverse reactions. The risk of adverse reactions was notably reduced if patients had both normal PA and CRP levels (PA >145.5 mg/L and CRP <102.23 mg/L) or only had either lower PA or higher CRP at one time. The incidence rate of visual abnormality was 30.00%, neurological abnormalities were 7.54%, and hepatotoxicity was 33.33% in our study. Older age and receiving intensive care were associated with a higher incidence rate of adverse reactions. Overall, the incidence rate of adverse reactions was 38.94% in our study. Patients who had lower PA and higher CRP at the same time tended to have a much higher incidence rate of adverse reactions (up to 70.27%). However, this study has some limitations, including the small number of patients and a simple research design, which require improvement in future studies.

## Conclusions

In conclusion, our study shed light on factors that can influence the concentration of VRZ, with particular attention to the often-overlooked parameter, PA, and the variable research results of CRP. We comprehensively screened multiple potential influencing factors, including biochemical indicators and combined medication, to identify the most influential factors affecting VRZ concentrations and overexposure. By utilizing the ROC curve, we determined the optimal cut-off points. Our findings highlight the significance of both PA and CRP in relation to VRZ concentrations and overexposure. Patients with PA <145.5 mg/L and CRP >102.23 mg/L simultaneously are at higher risk of VRZ overexposure and adverse reactions. This observation suggests that severe infections may compromise the safety of VRZ usage. For such patients, a dose-reducing regimen may be more advantageous in ensuring safe and effective treatment.
